# Supplementary figures and images for: Momordica charantia fruit reduces plasma fructosamine whereas stems and leaves increase plasma insulin in adult mildly diabetic obese Göttingen Minipigs
Source: PLoS One. 2024 Mar 18;19(3):e0298163. doi: 10.1371/journal.pone.0298163 (PMC10947704; doi:10.1371/journal.pone.0298163)

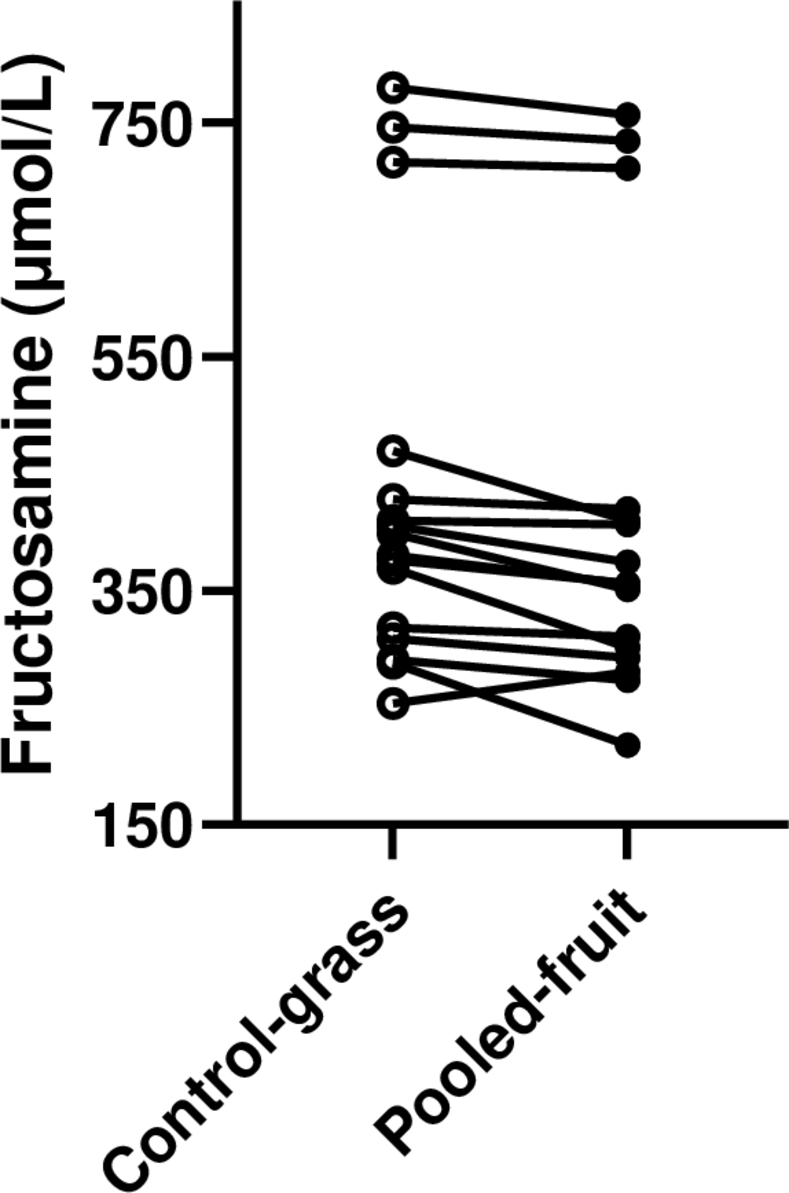

Supplement: S1 Fig — Plasma fructosamine concentrations were reduced (P = 0.002) by pooled fruit. (TIF) [file pone.0298163.s001.tif]

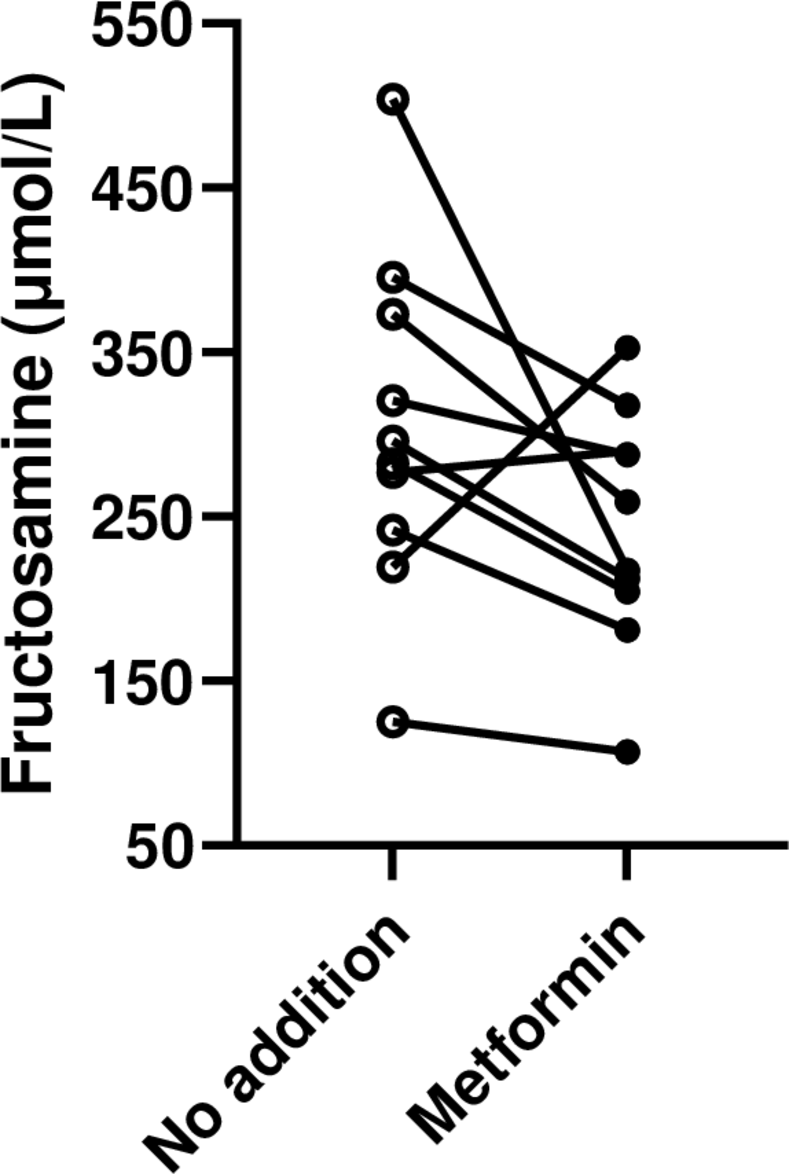

Supplement: S2 Fig — (TIF) [file pone.0298163.s002.tif]

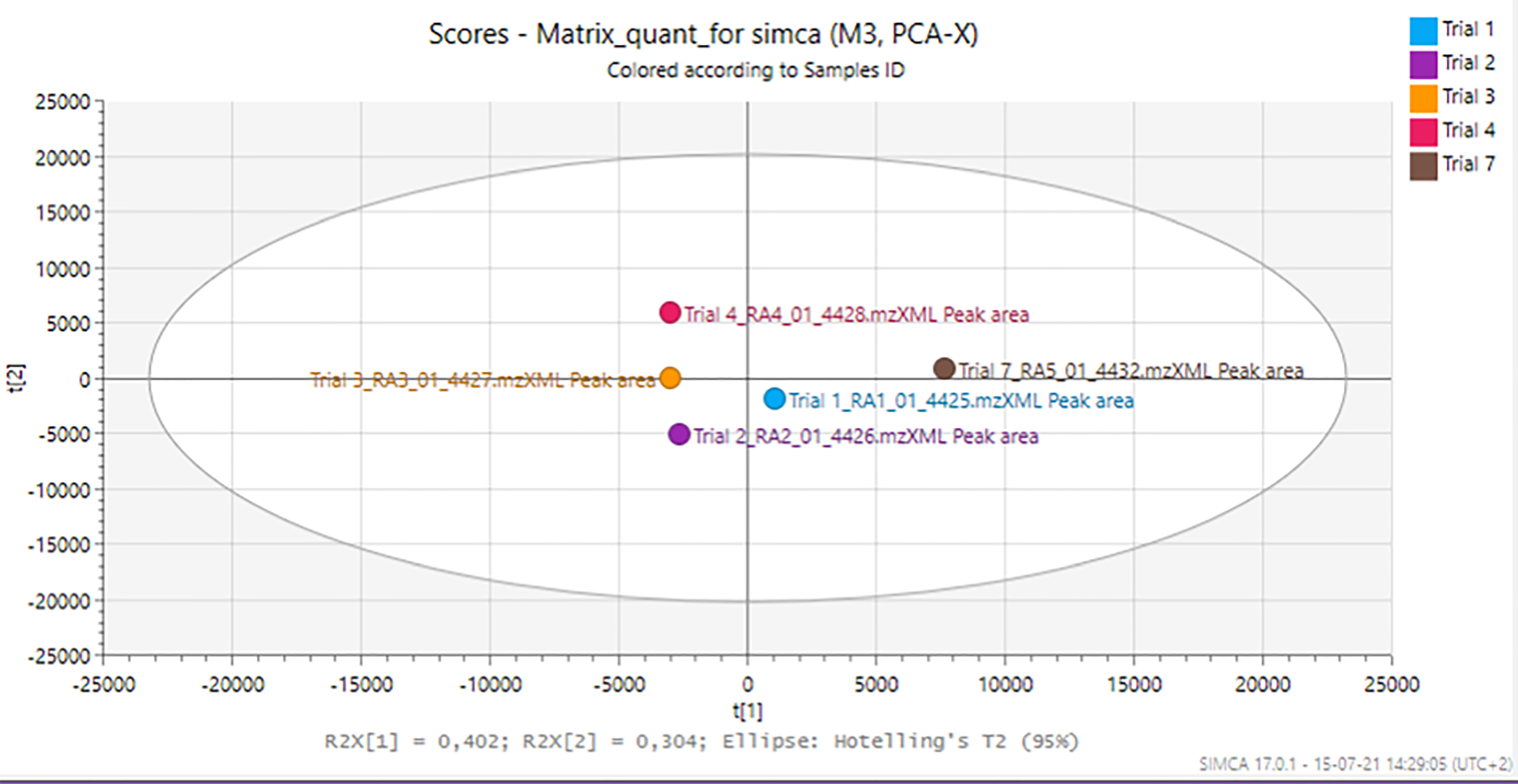

Supplement: S3 Fig — (TIF) [file pone.0298163.s003.tif]

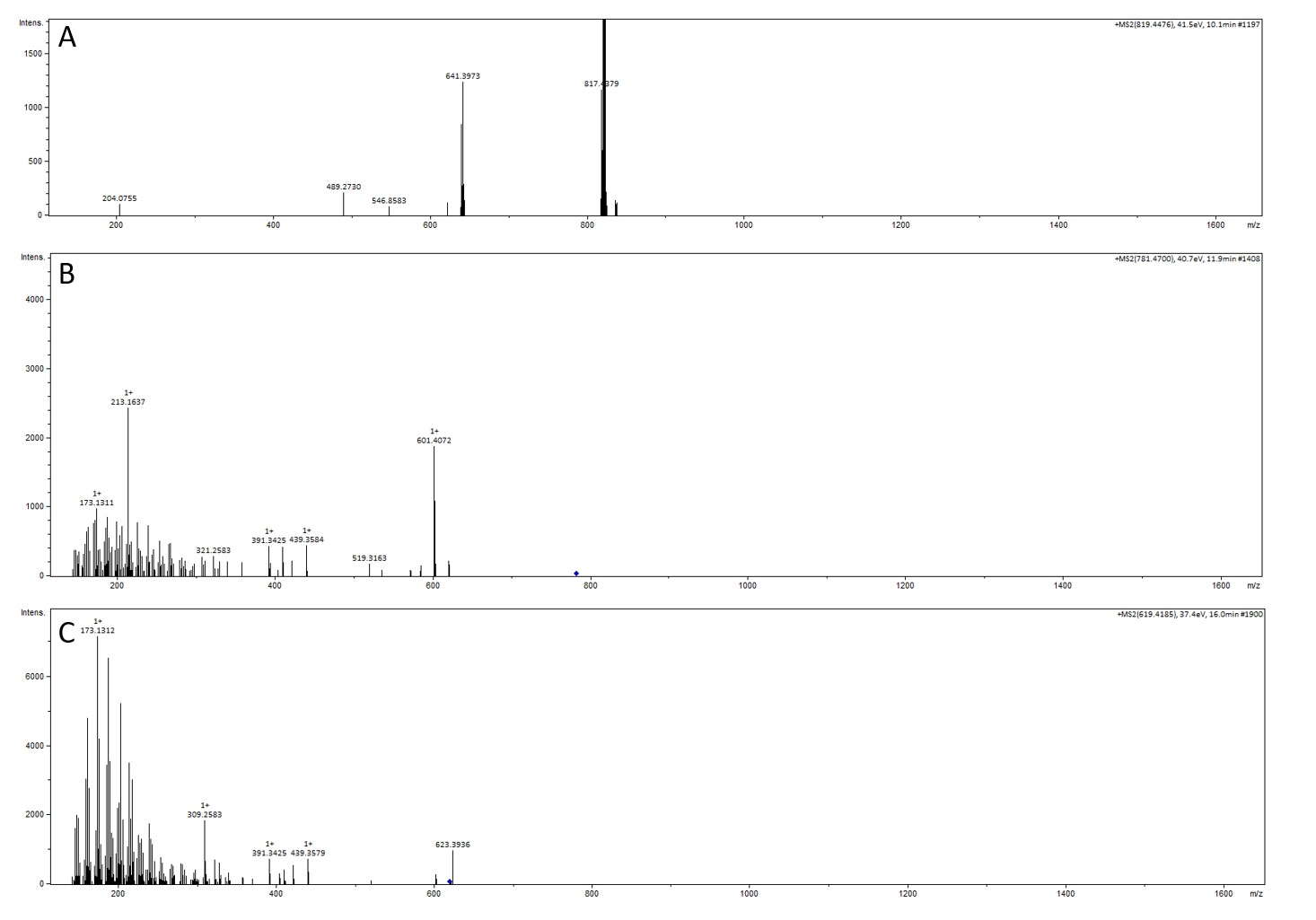

Supplement: S4 Fig — (TIF) [file pone.0298163.s004.tif]
