# Supplementary material for: Momordica charantia fruit reduces plasma fructosamine whereas stems and leaves increase plasma insulin in adult mildly diabetic obese Göttingen Minipigs
Source: PLoS One. 2024 Mar 18;19(3):e0298163. doi: 10.1371/journal.pone.0298163 (PMC10947704; doi:10.1371/journal.pone.0298163)
Supplement: S1 Table — (DOCX) [file pone.0298163.s005.docx]

**S1 Table. The individual and combined peak area’s under the curves (AUC in arbitrary units) for Xuedanoside H, Acutoside A and Karaviloside IX in Bitter gourd fruit from trials 1,2,3,4 and 7.**

| Trail Sample | Xuedanoside H | Acutoside A | Karavailoside IX | Total of the 3 saponins |
| --- | --- | --- | --- | --- |
| Wild type | 315742 | 102838 | 234963 | 653545 |
| HMT 242 | 69452 | 98961 | 146592 | 315006 |
| Palee | 188302 | 556759 | 978305 | 1723366 |
| Good healthy | 1721812 | 1633103 | 581468 | 3936384 |
| Bilai | 307166 | 30609 | 15881 | 353656 |
|  |  |  |  |  |
| Mean wild-type, HMT 242 and Bilai = 440735  Palee = > 3.9 fold increase  Good healthy = > 8.9 fold increase | | | | |
