# Supplementary material for: Momordica charantia fruit reduces plasma fructosamine whereas stems and leaves increase plasma insulin in adult mildly diabetic obese Göttingen Minipigs
Source: PLoS One. 2024 Mar 18;19(3):e0298163. doi: 10.1371/journal.pone.0298163 (PMC10947704; doi:10.1371/journal.pone.0298163)
Supplement: Attachment — Submitted filename: Response to reviewers.docx [file pone.0298163.s007.docx]

**Reviewer #1**: The manuscript represents an interesting study on the effect of Bitter gourd on a range of plasma compounds related to diabetes. The use of the diabetic pig model is highly relevant, and an impressing number of studies have been completed. The rationale for choosing mildly and overtly diabetic obese minipigs is missing and a group of obese non-diabetic minipigs would have been interesting as a control group. The chemical profiling of the Bitter gourd fruits is very interesting, however, the description of the methods is insufficient and the level of identification of the triterpenoids is not described. As these results are very interesting and the compounds are contributing to elucidating the mode of action of Bitter gourd, this should be given more focus – in the Introduction as well.

Answer: the following section has been added to the Introduction: “Therefore, the present study aimed to compare the effects of whole fruit or stems-leaves from five different Bitter gourd cultivars on metabolic control in adult diabetic obese Göttingen Minipigs, while concurrently conducting an in-depth analysis of triterpenoid compounds within these plant samples. The triterpenoid compounds were measured using a metabolomic approach that involved LC/MS-MS analysis, and the results were subjected to identification processes through systematic comparison, including fragment MS analysis, with the Dictionary of Natural Products database.”

Specific comments:

Line Comment

39 How was this confirmed. This statement is unclear.

Answer: These effects were statistically confirmed. Statistically has been added to the text.

75 Which compounds? Mode of action?

Answer: Compounds like glycosides, saponins, alkaloids and triterpenes. Mode of action is unclear because it consists of an undefined mixture of compounds. This has been added to the text.

Table 1 “Soja hulls RC 320-360” – is this a brand? Or what’s the reason for the name?

Answer: RC 320-360 means crude fibre 32-36%. This has been changed in Table 1.

144 Was stems and leaves not available for all cultivars?

Answer: Correct and the focus of the research was mainly on fruit.

Table 2 Please define “Crude fiber and non-starch polysaccharides” – maybe it should be including non-starch polysaccharides?

Answer: Indeed it is “Crude fibre including non-starch polysaccharides”. This has been changed in the table 2.

Table 2 Analyzed nutrient composition would have been better.

Answer: Agree. However the mild obesogenic diet and the dried grass were constant throughout the study and therefore we chose not to analyse the nutrient composition of the feed.

242 What’s the rationale for using Metformin?

Answer: Metformin is the most prescribed drug for the treatment of type 2 diabetes [19]. This has been added to the text.

272 How were the metabolites extracted?

Answer: The metabolites were extracted by chloroform or 80:20 methanol:water, which is described in the text of this paragraph.

273 No description of the GC-MS analysis.

Answer: The following was added to the text: “The samples were analyzed by a 7890A gas chromatograph equipped with a 7693 automatic sampler and a 5975C single quadrupole detector (Agilent, Folsom, CA, USA). Samples were separated on a DB-5 GC column (30 m x 0.25 mm, 0.25 µm film, J&W Science, Folsom, CA, USA) and eluted with He (99.9% purity) as a carrier gas at a flow rate of 1.5 mL.min^-1^. The oven temperature was programmed as follows: after an initial hold at 60 ^o^C for 1 min, temperature was increased at 7 ^o^C/min to 290 ^o^C and held for 5 min, then increased to 310 ^o^C at 5 ^o^C /min and held for a further 3 min. The injector was set at 280 ^o^C and 1 µL of the sample was injected in splitless mode. The interface temperature was 280 ^o^C, and the ion source and quadrupole temperature were 230 ^o^C and 150 ^o^C, respectively. The ionization energy in EI mode was 70 eV. Compounds were identified by comparison of their retention times and ion spectra with those of the pure compounds. Data was processed using Mass Hunter (B.07, Agilent), AMDIS (V. 2.63, Agilent), and MS search (V. 2.0, Agilent). Compounds were identified using NIST MS library (version 2008).”

319-321 Very short description, a bit more details would be nice. PCA-analysis?

Answer: The following was added to the text: “The obtained LC-MS data was further analyzed by a supervised multivariate data analysis, principal component analysis (PCA), in which dimensions of raw data were reduced one or two principal components (PC) in order to get clustering and to reveal and visualize the systematic variation within these compound profiles.”

331 Data could be presented in a table

Answer: Phenotype of the obese minipigs is now presented in a table.

Table 3 Please add that it is for all pigs – and the number of pigs per treatment.

Answer: The following was added to the Table: tested in all obese pigs combined (n=15).

Table 4 Same as for Table 3.

Answer: The following was added to the Table: tested in all obese pigs combined (n=20).

455 Why against “no addition of grass”?

Answer: Trial 8 without the addition of grass was discussed in lines 599 -611.

500 What is meant by “a more characteristic chemical pattern”? Please clarify.

Answer: “Compared to the result of GC-MS, LC-MS data showed more distinguished chemical profiling pattern”. This sentence is added to the text.

502 I don’t understand the interpretation of Figure 6. In my view trial 2 is very close to trial 3 and 4.

Answer: Regarding the interpretation of Figure 6, we acknowledge the reviewer's observation that trial 2 appears visually close to trials 3 and 4 in the PCA plot. While there may be visual proximity, it's important to clarify that this observation was based on the PCA results and coordinates as presented. In the context of our study and considering the available time constraints, we did not conduct additional pairwise statistical comparisons between these trials.

The PCA plot serves as a visual representation of the data, and while it suggests potential patterns and groupings, it does not provide definitive statistical conclusions. We recognize that further statistical analysis would be ideal to confirm the relationships between the trials more precisely. Given the current time limitations, we believe that presenting the PCA plot with the acknowledgement of its limitations is the most suitable approach within the scope of this study, because it allowed us to highlight compounds that were responsible for the differences in the samples. The relative concentration of these compounds highlighted by the PCA allowed us to identified differences between the sample.

We will include a clear statement in the revised manuscript, emphasizing that the PCA plot provides a preliminary indication of clustering but does not replace more detailed statistical analyses that might be conducted in future research.

Proposition of modification from lines 498 to 528:

However, our Liquid Chromatography–Mass Spectrometry (LC-MS) analysis, which was based on an 80:20 methanol:water extract of the Bitter gourd samples, yielded a more distinct chemical profile, particularly with regard to saponins. Through the quantification of compounds and subsequent Principal Component Analysis (PCA) (**Supporting Figure S3**), we observed clustering among the fruit samples used for pig trials 3 and 4 (cultivars “Palee” and “Good healthy”), which displayed improved in vivo glycemic control. Interestingly, these clusters were associated with specific peaks in the LC-MS chromatograms, suggesting the presence of cucurbitane triterpenoid glycosides that were highly specific to the Bitter gourd fruit cultivars used in trials 3 and 4.

These specific peaks, with retention times around 10.1 min (identified as Karaviloside IX), 11.96 min (identified as Acutoside A), and 16.03 min (identified as Xuedanoside H), were notably more abundant in samples from trials 3 and 4 compared to samples from trials 1, 2, and 7 (cultivars Wild-type, HMT 242, and Bilai) (**Figure 6**). Calculation of the combined peak areas under the curves (AUC) for Xuedanoside H, Acutoside A, and Karaviloside IX showed that these were increased in trial 3 (the cultivar “Palee”) more than 3.9-fold and in trial 4 (the cultivar “Good healthy”) more than 8.9-fold compared to the mean of the other trials (trial 1, 2, and 7, the cultivars Wild-type, HMT 242, and Bilai). AUC’s for Xuedanoside H, Acutoside A, and Karaviloside IX are shown in **Supporting Table S5).**

These findings suggest that a combination of these specific compounds, rather than a single compound, may be associated with the observed improvement in in vivo glycemic control in trials 3 and 4. The identification of these compounds was accomplished through a rigorous process involving the comparison of their mass-to-charge ratios (m/z) and the fragmentation patterns generated by MS/MS analysis. These data were meticulously matched with established databases, including the Dictionary of Natural Compounds and data from literature.

It is important to underscore that the identification of these compounds was made with a high level of confidence, bolstered by the precise matching of their mass spectra and fragmentation patterns to established reference data. Nevertheless, it remains imperative to acknowledge that further research is essential to unveil the precise mechanisms and interactions of these compounds in relation to glycemic control. This comprehensive understanding will require additional investigations, potentially encompassing bioactivity assays, metabolic studies, and mechanistic experiments, to corroborate their roles in mediating the observed improvements in in vivo glycemic control during trials 3 and 4.

Figure 6 could be exchanged for Supporting Figure S3, which is far more interesting.

Answer: Figure 6 has been exchanged for **Supporting Figure S3.**

505 What is meant by “This classification”?

Answer: The sentence has been rephrased, see proposition above for lines 498-528.

514 The details on this analysis are missing in Materials and methods.

Answer: The identification was based on the fragmentation pattern and molecular adduct ion from the same LC-MS data. The sentence has been rephrased, see proposition above for lines 498-528.

518 How were the triterpenoids identified? Authentic standards, fragmentation pattern ….? What is the level of identification?

Answer: The identification was based on the fragmentation pattern and molecular adduct ion of LC-MS data (see answer above).

Table 7 Move to supplementary material.

Answer: Table 7 has been moved to supplemental material as **Supporting Table S4.**

551 Has these triterpenoids previously been identified in Bitter gourd?

Answer: Five references have been added to the list.

Xuedanoside H 🡪 cucurbitane found in another Cucurbitaceae, Hemsleya amabilis :

[24] Jin Y, Zhou X, Yang J, Xu X, Zhang J, Ma G. Bioactive triterpenoid saponins from the tubers of Hemsleya amabilis Diels. Fitoterapia. 2019 Nov 1;139:104404. <https://doi.org/10.1016/j.fitote.2019.104404>

[25] Li Z, Chen M, Chen F, Li W, Huang G, Xu X, Wang S, Ma G, Cui P. Cucurbitane triterpenoid entities derived from Hemsleya penxianensis triggered glioma cell apoptosis via ER stress and MAPK signalling cross-talk. Bioorganic Chemistry. 2022 Oct 1;127:106013. <https://doi.org/10.1016/j.bioorg.2022.106013>

Karavilosides are described in Momordica charantia :

[26] Perera WH, Shivanagoudra SR, Pérez JL, Kim DM, Sun Y, K. Jayaprakasha G, S. Patil B. Anti-inflammatory, antidiabetic properties and in silico modeling of cucurbitane-type triterpene glycosides from fruits of an Indian cultivar of Momordica charantia L. Molecules. 2021 Feb 16;26(4):1038. [10.3390/molecules26041038](https://doi.org/10.3390/molecules26041038).

Acutoside A 🡪 found in different plant families, including Cucurbitaceae like Luffa acutangular:

[27] Nagao T, Tanaka R, Iwase Y, Hanazono H, Okabe H. Studies on the constituents of Luffa acutangula Roxb. I. Structures of acutosides AG, oleanane-type triterpene saponins isolated from the herb. Chemical and pharmaceutical bulletin. 1991 Mar 25;39(3):599-606. <https://doi.org/10.1248/cpb.39.599>

This paper helped us a lot for the identification of Karaviloside and Acutoside A : Metabolite Profiling and Quantitation of Cucurbitacins in Cucurbitaceae Plants by Liquid Chromatography coupled to Tandem Mass Spectrometry :

[28] Ul Haq F, Ali A, Khan MN, Shah SM, Kandel RC, Aziz N, Adhikari A, Choudhary MI, ur-Rahman A, El-Seedi HR, Musharraf SG. Metabolite profiling and quantitation of cucurbitacins in Cucurbitaceae plants by liquid chromatography coupled to tandem mass spectrometry. Scientific Reports. 2019 Nov 5;9(1):15992. [10.1038/s41598-019-52404-1](https://doi.org/10.1038%2Fs41598-019-52404-1)

560 Please reformulate this sentence.

Answer: The sentence “The result is that after a meal less glucose is generated and the transport of glucose from the gut to blood is reduced” has now been transformed in: “Impaired degradation of starch leads to a lower influx of glucose from the gut to blood, thereby reducing blood glucose concentrations”.

581-584 Very speculative – reformulate or provide evidence.

Answer: Line 581-584: “These effects seem small but in combination with changes in life style (reduction in food intake and increase in physical activity) and in combination with anti-diabetic medication, the effects may be partly cumulative.” Evidence is presented in the following sentence where it is stated that the mode of action of Bitter gourd is different from Metformin and thus te effects could be additive. “Indeed, with respect to mono-therapy using the purified anti-diabetic drug metformin, the mode of action of Bitter gourd fruit seems to be different from metformin. Metformin is known to reduce hepatic glucose production which is the primary contribution to the blood glucose-reducing effect by metformin; however, the complete mechanism of action of metformin is still not fully understood [27,28].” The following sentence has now been added: “By contrast, Bitter gourd fruit is known to enhance cellular glucose uptake and inhibit glucose absorption from intestine [1]. Yet, to have proof for the possible additive effects of Bitter gourd and metformin, further studies are needed.”

590-593 Why wasn’t this pig excluded?

Answer: We could not detect any experimental mistakes with respect to the measurements related to this pig and the pig was eating well and had no clinical signs of illness.

597-598 Speculation.

Answer: Indeed, also see answers to 581-584. We feel that multiple approaches (strategies/treatments), each having their own “mode of action”, may be cumulative in their abilities to improve glycemic control in mildly diabetic obese subjects.

605 Could the difference in dietary fiber play a role?

Answer: Yes that is possible. A difference in fibre load may also contribute to an effect on plasma fructosamine concentrations. Fibre is known to affect glucose metabolism [41]. There are many different fibre-types so it is hard to predict the net effect of fibre in Bitter gourd or in grass (as control) on glycemia. This text has been added at line 611.

607-608 Can these percentages be added in this way?

Answer: It is a correction based on previous studies [40]. The inverse correlation between meal size and glycemic control is linear for the size (4%) of correction.

619 Were there any comparable effects? Discuss the animal models.

Answer: Yes, “Most studies showed a reduction of hyperglycemia in diabetic rodents”. This sentence has been added to the text. The numerous (>10) animal models using diabetic rats and mice described in over 100 studies have been summarized and discussed in [1-3,5]. We feel it would be better to refer to these reviews than to repeat their discussions.

645-648 This is very speculative.

Answer: Agree. However, any dietary treatment which elevates plasma insulin concentrations in subjects which could benefit from an increase in plasma insulin concentrations is a topic for further investigations.

The text was adjusted as follows: A recent study by us [43] showed that Bitter gourd stems and leaves at inclusion levels of 0.65-1.3% in feed of growing pigs had no effects on plasma insulin and urea concentrations and on pig performance. These inclusion levels of Bitter gourd stems and leaves in feed were however 3-fold to 6-fold lower compared to the present study (4% inclusion level). Any possible beneficial effects of Bitter gourd stems and leaves in Livestock production require further investigation.

650-664 This section could be moved to the beginning of the Discussion.

Answer: We have moved this section to the beginning of the Discussion.

**Reviewer #2:**

119 Why did you choose female pigs?

Answer: Female pigs were chosen because they are more sensitive to metabolic abnormalities than males [Christoffersen BO, Grand N, Golozoubova V, Svendsen O, Raun K. Gender-associated differences in metabolic syndrome-related parameters in Göttingen minipigs. Comp Med. 2007 Oct;57(5):493-504. PMID: 17974133]. This has been added to the text and references.

Are the females pregnant or nulliparous?

This choice should be justified

Laboratory animals, especially females, are more sensitive than males and when females are pregnant or nulliparous, the tests may be influenced by these physiological characteristics. These are some of the reasons why it is important to specify the physiological characteristics of the animals tested

Answer: Multiparous, non-pregnant sows were used to reflect adult women. This has been added to the text.

304 It should be remembered that diabetes is not limited to the measurement of blood sugar but also to the measurement of triglycerides which are made up of lipids and therefore form fat. As it happens, we can know that in the Krebs cycle, there is a gluconeogenesis, which form glucose from non-carbohydrate elements. The fat that is formed as a reserve is transformed and takes on the connotation of sugar ready to be used by the brain to continue feeding the body in case of prolonged fasting. In summary, a diabetic needs to control both sugar and membrane lipids and to have at his disposal foods such as bitter gourd to slow down the frequent desire of appetite in order to control the two parameters mentioned above.

Answer: We fully agree with your statement. That is why we have measured blood triglycerides as well.

I congratulate the authors for the quality and the scope of the study which reinforces the endogenous knowledge and moreover relieves the patients of this chronic pathology which gnaws rich and poor as well in Africa in Europe etc..

Answer: Thank you!

**Reviewer #3:** Here are some suggestions to improve the quality of the manuscript

Abstract

1. The methodology needs to be rephrased for clarity. How many groups? What did each group receive?

Answer: The methodology section has been adjusted as follows: Twenty streptozotocin-induced diabetic (D) obese Minipigs (body weight ~85 kg) were subdivided in mildly and overtly D pigs…….. The treatments were described in the last sentence of the background section: “The present study compared the effects of whole fruit or stems-leaves from five different Bitter gourd cultivars on metabolic control in human-sized adult diabetic obese Göttingen Minipigs”.

2. Did you give the same treatment to both mildly and overtly diabetic obese minipigs?

Answer: Yes, see lines 32-35 and Figure 1.

3. How many cultivars of bitter gourd did you use?

Answer: Five, see line 30.

Methods

1. Line 119, what is the total number of pigs used? How many are diabetic?

Answer: Twenty out of 24 pigs were used as described in lines 174-215. Nineteen were diabetic and 1 reference non-diabetic control.

2. Lines 231-242, why did you exclude cultivars HMT 242, Good health, and Palee versus grass for better gourd stems and leaves?

Answer: These stems and leaves were not available from Vietnam.

3. How many pigs did you use in each trial?

Answer: That varied among trials as indicated in lines 105-107 and indicated in the various Tables: The number of pigs available per strategy group and per trial varied among trials depending on the occurrence of incomplete meal intake or any missing blood samples.

4. Why did you choose grass as the control diet for some trials and no supplement as a control for other trials?

Answer: To test the effect of nutritional load. Please see lines 599-611 for discussion.

5. Line 272, did you use a single extract? If no, state all the extracts used.

Answer: The following text was added following line 272: “The extracts for the GC/MS analysis were prepared using MeOH and n-hexane. For MeOH: Extract 30 mg of powder (15 min ultrasonication) with 1 mL of MeOH and aliquot by 100 µL. Remove the solvent with speed vacuum, take one of the aliquot and add 100 µL of pyridine containing 1 mg/mL of methyl palmitate, Add 100 µL of BSTFA with 1% TMCS and react for 60 min at 80°C in a heating block. For n-hexane: 30 mg of dried powder in 1.0 mL of hexane containing 0.5 mg of methyl palmitate, (15 min ultrasonication) and Centrifugation (10 min), Take 200 µL from the supernatant for the analysis. The extract for HPTLC was prepared as follows: 30 mg of dried powder in 1.0 mL of acetone:n-hexane (1:1) extraction and n-hexane extraction and centrifugation.”

6. Did you perform each trial on both mildly and overtly diabetic obese minipigs?

Answer: Yes, see question 2 of the abstract..

Results

1. Lines 302-321, did you compare the insulin, cholesterol, blood glucose, and fructosamine level between the mildly and overtly diabetic obese minipigs?

Answer: Yes we did, please see the newly added Table 3

2. Lines 332-352, please compare the fructosamine, insulin, cholesterol, LDL, HDL, triglycerides, and ketones between obese minipigs without streptozotocin treatment (control), mildly and overtly diabetic obese minipigs using the significant increase or decrease.

Answer: We have presented the phenotype of the mildly and overtly diabetic pigs including a reference non-diabetic pig in Table 3. We have not conducted statistics but showed mean values compared to one reference pig.

3. Indicate if there are significant changes in all the parameters evaluated between the control and treatment groups in all the trials.

Answer: All significant changes have been mentioned in the results section. If parameters were not mentioned, there was no significant difference between the control and treatment groups for that parameter.

4. Use symbols to indicate significant changes in tables 3-6.

Answer: We have added * as P<0.05.

5. The results for glucosamine, blood glucose, and insulin should be presented for all the cultivars.

Answer: We chose to present only results which differed significantly. By presenting all results on plasma fructosamine, blood glucose and plasma insulin, i.e. for all 10 trials including separately all pigs combined, mildly diabetic pigs and overtly diabetic pigs, a tremendous amount of text needs to be added to the results section which reduces the readability of the paper. We prefer not to show the non-significant results but instead, all results can be found in the supplemental file.

**Reviewer #4:** The manuscript entitled “ fruit reduces plasma fructosamine whereas stems and leaves increase plasma insulin in adult mildly diabetic obese Göttingen Minipigs.” has been reviewed. The authors present a very interesting work on the use of Bitter gourd and the potential use as alternative medicine for glucose and obesity control.

The animal study was complex but carefully designed and the authors have a good experience in establishing swine models for metabolic diseases, particularly the diabetic induced minipig model.

The authors have done a good job presenting the results and implications of their findings. However, several of the finds particularly to the compounds which can be related to the positive effects of the Bitter gourd should be better clarified.

Minor comments

Line 31: the term human-sized adult GM is rather confusing. Although it’s understandable what the authors would like to say, that the model of GM is representative of an adult human, human-sized minipig is not fully correct. Please rephrase. Similar for line 83.

Answer: The term human-sized has now been omitted.

Line 42: metabolomics” is too general. One could say “studied by metabolomics phenotyping” or “metabolomics profiling”.

Answer: Metabolomics has been changed in “metabolomics profiling”.

Line 550, Line 669: it would more advised to refer to these compounds in term of “compound intensities” rather than concentration since they were not actually quantified using chemical standards.

Answer: We have changed the term in “compound intensities” and have now omitted the use of concentration.

Major comments on section Section 492: Chemical profiling

Line 499. There is a discrepancy between what is written here and what was presented in the materials and menthods section. Line 499 mentions 80:20 methanol:water whereas in the M&M the LC-MS extractions were done using 100% methanol. Which one is correct?

Answer: 80:20 methanol:water is the good one. This has now been adjusted in the M&M section.

Please provide more information how extracts were prepared for the LCMS analysis. The authors only mention the extraction solvent used and nothing on the sample preparation.

Answer: We now have added more details to the M&M section ‘Thirty mg of dried and powdered samples were ultrasonicated for 20 min with 1 mL of 80% methanol and centrifuged for 20 min to obtain a clear supernatant (13000 rpm). The samples were filtered by 0.2 mm membrane filter.`

Line 501: PCA analysis was performed on the data but there is no mention of how the metabolomics data was extracted, what software was used and how was the data treated before PCA analysis. Please add this information in M&M.

Answer: We now have added more details to the M&M section Statistical and data analyses ‘Data files obtained from the LC–MS/MS analyses were converted to mzXML format using Brucker Daltonics DataAnalysis (version 4.1, Bremen, Germany). The LC-MS/MS data was processed using MZMine2 (Pluskal et al., 2010). To build the feature matrix, mass detection was performed using centroid data. The noise level was set at 10,000 for MS and 100 for MS/MS. The chromatograms were built using the ADAP chromatogram builder (Myers et al., 2017) with a minimum number of scans of 3, group intensity threshold 1000, minimum highest intensity 10,000 and m/z tolerance of 0.05. Chromatograms were deconvoluted using a baseline cutoff algorithm with the following parameters: minimum peak height of 10,000, peak width range of 0.02–1.00 min, and baseline level of 1000. Chromatograms were deisotoped using an isotopic peak grouperalgorithm with an m/z tolerance of 0.05 and retention time tolerance of 0.1 min. The features of each sample were aligned using a join alignment algorithm with the following parameters: 0.05 m/z tolerance and 0.1 min retention time tolerance.

Besides the following references were added to the M&M section above:

[22] Pluskal T, Castillo S, Villar-Briones A, Orešič M. MZmine 2: modular framework for processing, visualizing, and analyzing mass spectrometry-based molecular profile data. BMC bioinformatics. 2010 Dec;11(1):1-1.

[23] Myers OD, Sumner SJ, Li S, Barnes S, Du X. One step forward for reducing false positive and false negative compound identifications from mass spectrometry metabolomics data: new algorithms for constructing extracted ion chromatograms and detecting chromatographic peaks. Analytical chemistry. 2017 Sep 5;89(17):8696-703.

Line 501: Please note that the score plot of the PCA analysis shows that the Sample from trial 2 is also clustering rather close with samples from Trial 4 and Trial 3 and separating from Trial 7 and Trial 1 along the first Principal component (PC1).

Answer: Based on a similar comment from reviewer 1 we rephrased this. See answer already given to reviewer 1.

Line 501: Is it correct that the authors mention quantification? Perhaps they meant compound identification. Quantification implies the use of standard curves and the use of internal standards in the analysis which is not mentioned in the materials and methods.

Answer: We agree to the reviewer’s comments. Compound quantification has been changed in compound identification.

Line 518: How was the identification preformed? Was it done by comparison of spectra to that of metabolomics databases ? If so which databases were used?

Answer: The identification of the saponins was based on previously reported data. For added references please see reviewer 1.

Please provide in supplemental material the fragmentation spectra of these specific compounds.

Answer: The fragmentation spectra have been added as **Supporting Figure S4**

**Karaviloside IX, C_42_H_68_O_14_ - Peak at 10,1 min– 819,44618**

**m/z 819,44618 [M+Na]+, MS/MS 641.3973**

**Acutoside A, C_42_H_68_O_13,_ - Peak at 11,96min– 781.46805**

**m/z 781,46805 [M+H]+, MS/MS 601.4072**

**Xuedanoside H, C_36_H_58_O_8_ - Peak at 16,03min– 619,41571**

**m/z 619,41571 [M+H]+, MS/MS 439.3579**

Supporting Figure S4. The fragmentation spectra of Karaviloside IX, Acutoside A and Xuedanoside H.

Furthermore, the authors mention identification. To which level according to Sumner et al were identified? Level 1 using chemical standards to compare, or only annotations based on similarities to public mass spectra databases.

Please mention this, as it is important to have a better understanding of the level of accuracy in the identification of molecular features using metabolomics. Otherwise wrong conclusions can be drawn because of poor compound annotations.

Sumner LW, et al. Proposed minimum reporting standards for chemical analysis Chemical Analysis Working Group (CAWG) Metabolomics Standards Initiative (MSI). Metabolomics. 2007 Sep;3(3):211-221. doi: 10.1007/s11306-007-0082-2. PMID: 24039616; PMCID: PMC3772505.

Answer: Identification is conducted by annotations based on similarities to public mass spectra databases (Sumner et al, 2007).

The Sumner reference has been added to the list:

Sumner LW, Amberg A, Barrett D, Beale MH, Beger R, Daykin CA, Fan TW, Fiehn O, Goodacre R, Griffin JL, Hankemeier T. Proposed minimum reporting standards for chemical analysis: chemical analysis working group (CAWG) metabolomics standards initiative (MSI). Metabolomics. 2007 Sep;3:211-21.

Line 513: how was this analysis preformed more “focused”? Please clarify.

Answer: The paragraph was rephrased (see answers to reviewer 1).

Journal Requirements:

When submitting your revision, we need you to address these additional requirements.

1. Please ensure that your manuscript meets PLOS ONE's style requirements, including those for file naming. The PLOS ONE style templates can be found at

<https://journals.plos.org/plosone/s/file?id=wjVg/PLOSOne_formatting_sample_main_body.pdf> and

[https://journals.plos.org/plosone/s/file?id=ba62/PLOSOne_formatting_sample_title_authors_affiliations.pdf](https://eur03.safelinks.protection.outlook.com/?url=https%3A%2F%2Fjournals.plos.org%2Fplosone%2Fs%2Ffile%3Fid%3Dba62%2FPLOSOne_formatting_sample_title_authors_affiliations.pdf&data=05%7C01%7Csietsejan.koopmans%40wur.nl%7Cf27391f92b474da9a5b608dbab3dc42d%7C27d137e5761f4dc1af88d26430abb18f%7C0%7C0%7C638292054492003685%7CUnknown%7CTWFpbGZsb3d8eyJWIjoiMC4wLjAwMDAiLCJQIjoiV2luMzIiLCJBTiI6Ik1haWwiLCJXVCI6Mn0%3D%7C3000%7C%7C%7C&sdata=lhdoVtiELby5YCwjWDYygwPZDQKrT2M8mfBfd%2BcLzLg%3D&reserved=0)

Answer: The PLOS ONE style has been checked and adjusted when necessary. These adjustments were made in the manuscript and not in the manuscript with track changes to avoid unreadability.

2. Please include your full ethics statement in the ‘Methods’ section of your manuscript file. In your statement, please include the full name of the IRB or ethics committee who approved or waived your study, as well as whether or not you obtained informed written or verbal consent. If consent was waived for your study, please include this information in your statement as well.

Answer: The following text was added to the `Methods’ section: Experimental protocols describing the management, surgical procedures, and animal care were reviewed and approved by the ASG-Wageningen Animal Care and Use Committee (Wageningen, The Netherlands). AVD license number 40100201858.

3. Please upload a new copy of Figure 6 as the detail is not clear. Please follow the link for more information: <https://blogs.plos.org/plos/2019/06/looking-good-tips-for-creating-your-plos-figures-graphics/>" <https://blogs.plos.org/plos/2019/06/looking-good-tips-for-creating-your-plos-figures-graphics/>

Answer: Figure 6 has now been adjusted and re-uploaded as Supporting Figure S3 as suggested by reviewer 1.

Additional Editor Comments:

I have additional comment as follows:
The authors need to go deeper into the study by investigating the molecular mechanisms behind their findings.

Answer: the molecular mechanisms behind glycemic control have been further discussed based on the various questions and suggestions by the reviewers.
